# Multimodular Ultrasound Orientation: Residents’ Confidence and Skill in Performing Point-of-care Ultrasound

**DOI:** 10.7759/cureus.3597

**Published:** 2018-11-15

**Authors:** Lori A Stolz, Richard Amini, Elaine Situ-LaCasse, Josie Acuña, Steven C Irving, Lucas Friedman, Albert B Fiorello, Nicholas Stea, Heinrich Fan, Srikar Adhikari

**Affiliations:** 1 Emergency Medicine, University of Cincinnati, Cincinnati, USA; 2 Emergency Medicine, University of Arizona, Tucson, USA; 3 Emergency Medicine, University of California, Riverside, USA

**Keywords:** point-of-care ultrasound, residents, emergency medicine, orientation course, multimodular

## Abstract

Introduction

The objectives of this study were to determine if a multimodular introductory ultrasound course improved emergency medicine intern confidence in performing a point-of-care ultrasound and if our educational objectives could be met with our chosen structure.

Methods

This is a prospective, observational study evaluating three consecutive incoming emergency medicine residency classes from three residency programs. A one-day introductory ultrasound course was delivered. The course consisted of 1) flipped classroom didactics, 2) in-person, case-based interactive teaching sessions, and 3) check-listed, goal-driven, hands-on instruction.

Results

Over three years, 73 residents participated in this study. There was no significant difference in performance on the written test (p = 0.54) or the skills assessment (p = 0.16) between years. Performance on the written pre-test was not a predictor of performance on the skills test (R^2 ^= 0.028; p = 0.19). Prior to training, residents were most confident in performing a focused assessment with sonography for trauma examination (median confidence 5.5 (interquartile range (IQR): 3 - 7) on a 10-point Likert scale where 1 represents low confidence and 10 represents high confidence). They reported the lowest confidence in performing a cardiac ultrasound (3 (IQR: 2 - 6)). Following training, residents reported increased confidence with all applications (p < 0.001). Eighty-five percent (confidence interval (CI): 73, 92) of residents agreed that the online ultrasound lectures effectively teach point-of-care ultrasound applications and 98% (CI: 88, 100) agreed that case-based interactive sessions helped them understand how ultrasound changes the management of acutely ill patients.

Conclusions

A written test of knowledge regarding the use of point-of-care ultrasound does not correlate with procedural skills at the start of residency, suggesting that teaching and evaluation of both types of skills are necessary. Following a multimodular introductory ultrasound course, residents showed increased confidence in performing the seven basic ultrasound applications. Residents reported that an asynchronous curriculum and case-based interactive sessions met the learning objectives and effectively taught point-of-care ultrasound applications.

## Introduction

Point-of-care ultrasound performance and interpretation is a required patient care competency that must be taught and assessed in emergency medicine residency programs [1-2]. As residency programs shape their curricula, one common challenge they face is initiating ultrasound instruction to residents at the beginning of their training. Medical school instruction in point-of-care ultrasound instruction varies widely with only 62% including point-of-care ultrasound in their curriculum [3]. Of the programs that do offer point-of-care ultrasound education in the curriculum, the student experience varies greatly with some providing longitudinal integrated curricula over four years and others offering instruction-only during the electives or the medical student emergency medicine rotation [4-9]. Programs with structured ultrasound curricula graduate medical students with significantly improved ultrasound skill levels compared to medical schools who do not implement a formal curriculum in point-of-care ultrasound [10].

While efforts are underway to establish uniform standards for instruction of ultrasound in medical student education, residency programs are tasked with providing instruction to all incoming residents regardless of prior training to ensure that each resident has a foundation of ultrasound machine use and clinical ultrasound performance [11]. This is often started in an ultrasound orientation or introductory course offered early in residency training in the month of July [12]. It is recommended that this is delivered as a one-day course that covers basic ultrasound applications, physics, and knobology and includes didactic as well as hands-on sessions [13]. As residency programs across the United States are simultaneously addressing this need at their respective sites, there is value in learning from the experience of other educators at various institutions in how to best provide this education. The constraints of resident and faculty time and resources of the residency program limit what is possible to teach within these orientation sessions. Despite the recommendation to provide these sessions and the nearly ubiquitous implementation in emergency medicine residency programs, it is unclear if these orientation sessions improve resident confidence in performing an ultrasound, written or technical skills, or frequency of use on later clinical shifts.

In response to these challenges, we developed a multimodular introductory ultrasound course for incoming residents with three main components: 1) flipped classroom didactics, 2) in-person, case-based interactive teaching sessions, and 3) check-listed, goal-driven, hands-on instruction with assessments. We structured our introductory ultrasound course to maximize available resources to teach residents. We focused on the application of clinical decision-making using point-of-care ultrasound with less emphasis on in-person “how-to” didactic lectures and goal-directed hands-on sessions. This style of introductory ultrasound course was chosen as a way to provide learning to residents with advanced skills and minimally required skills simultaneously.

We present here the details of our introductory ultrasound course in order to share our experience with other academic institutions who are also delivering ultrasound orientation sessions. The objectives of this study were to determine if a multimodular introductory ultrasound course improved emergency medicine resident confidence and if our educational objectives could be met using our chosen structure.

## Materials and methods

Study design and population

This was a prospective observational study. The study took place with residents from three residency programs: two emergency medicine residency programs and a combined pediatric-emergency medicine residency program. This introductory ultrasound course was implemented for three consecutive years. This study was approved as exempt by the University of Arizona Institutional Review Board. The residents’ pre-test scores, their hands-on skills assessment scores, and a survey of the perception of their ultrasound performance confidence levels were recorded as anonymized data. A one-day introductory ultrasound course was delivered with the following components: 1) asynchronous learning of various ultrasound applications prior to ultrasound orientation; 2) a written knowledge assessment; 3) interactive sessions focused on patient cases and clinical integration of point-of-care ultrasound; 4) goal-driven, hands-on skills instruction using checklists; 5) an observed assessment of technical skills; and 6) a survey of resident perception.

Asynchronous learning

One week before the one-day orientation session, the course participants received an email with the asynchronous learning materials. The Academy of Emergency Ultrasound narrated lectures series on physics, extended focused assessment with sonography for trauma (E-FAST) examination, aorta, renal, thoracic, and echocardiography were sent, as well as links to vascular access and skin and soft tissue lectures [14-15]. Narrated lectures were reviewed by ultrasound faculty for content, clarity, and relevance prior to selection. Institution-specific portable document format (PDFs) detailing departmental ultrasound policies, system workflow, instructions on probe care, and ultrasound examination documentation were also e-mailed to the residents. The estimated time to review the asynchronous learning materials was approximately four hours.

Written knowledge assessment

On the day of the course, the residents’ baseline knowledge of ultrasound was assessed with a written 37-question pre-test, including items related to basic ultrasound physics, system workflow, anatomy, and pictorial questions that would test their ability to recognize artifacts, pathology, and appropriate ultrasound settings. This written assessment was reviewed by multiple faculty members for relevance and completeness.

Case-based didactic lectures

The ultrasound-trained emergency medicine faculty organized two and a half hours of interactive sessions. These were case-based and focused on the clinical integration of point-of-care ultrasound. A short review of physics concepts, departmental ultrasound policies, and requirements of the required two-week postgraduate year (PGY)-1 ultrasound rotation were also included. Instruction on how to perform basic scans was not covered as it was covered in the asynchronous curriculum.

Hands-on educational session

After the lectures, the residents participated in hands-on teaching using live models with either a Philips Sparq (Royal Philips, Amsterdam, Netherlands), Philips CX50 (Royal Philips, Amsterdam, Netherlands), Zonare Z-One (Mindray, Mahwah, NJ), Zonare ZS3 (Mindray, Mahwah, NJ), Mindray M9 (Mindray, Mahwah, NJ), or Mindray TE7 (Mindray, Mahwah, NJ) ultrasound machine. During the hands-on portion of the course, emergency ultrasound faculty members and fellows taught machine use, image optimization, and seven basic point-of-care ultrasound applications: focused assessment with sonography for trauma (FAST), cardiac, renal, aorta, soft tissue, thoracic, and vascular access. Groups were limited to four participants with one faculty member to maximize each participant's time using the machine and manipulating the probe. Instructors utilized a standardized skills checklist to ensure that equivalent information was delivered between groups and that the sessions were goal-directed and streamlined. Checklists were developed and reviewed for completeness and relevance by multiple faculty members.

Hands-on skills assessment

Following this hands-on education, the participants’ skills were assessed using an objective structured clinical examination-style assessment. Residents were directly observed by faculty members or fellows other than those who had instructed them and rated on their machine use, image acquisition, and image optimization in real time using live models. A series of endpoints on a 5-point scale with 1 being “Below expectation,” 3 being “Meets expectations,” and 5 being “Exceeds expectations” were utilized for assessment. They were asked to demonstrate workflow steps on the machine (entering an order, selection of the appropriate probe and preset, etc.), appropriate image acquisition, and image optimization. No feedback was provided to the participants until the assessment was completed. Afterward, faculty discussed with each resident their performance and offered immediate feedback.

Survey of resident opinion

The residents’ last assignment of the day was to complete a survey asking their prior number of ultrasound examinations performed, rating their confidence level in the basic ultrasound applications that were taught during the course, their opinion of the methods of educational delivery, and whether the educational objectives were met.

Data analysis

All statistical analyses were done using Stata, version 14 (Stata Corp, College Station, TX). Means (95% confidence intervals (CIs)), medians (interquartile range - IQR), and percentages (95% CIs) were used for descriptive data. Categorical data between the groups were analyzed using Fisher’s exact test. Differences between groups for continuous data were tested using the Student’s t-test (paired and unpaired, as appropriate) or the one-way analysis of variance (ANOVA) for more than two groups for parametric data. The Kruskal-Wallis rank test or Wilcoxon matched-pairs signed rank test was used for non-parametric data. An alpha of 0.05 was used for statistical significance. To assess normality for continuous data, we used the Shapiro-Wilk test for normal data, and to test the assumption of equal variances between groups, we used Bartlett’s test for equal variances. We used linear regression to determine if higher pre-test scores correlated with higher skills assessment scores.

## Results

Over three years, 73 residents participated in the introductory ultrasound course [16]. There was no significant difference in performance on the written test (p = 0.54) or the skills assessment (p = 0.16) between years. Residents averaged 78.0% (95% CI: 75.8, 80.3) on the pre-test and 72.8% (95% CI: 69.9, 75.6) on the skills assessment. Performance on the pre-test was not a predictor of performance on the skills test (R^2^ = 0.028; regression coefficient = 0.21; 95% CI: -0.11, 0.53; p = 0.19). Mean skills assessment score was related to faculty evaluator (ANOVA global p-value = 0.0038) with mean skills assessments score of each grader ranging from a low of 58.7% to a high of 86.7% with a median skills assessment of 71.6 (IQR: 66.3 - 74.3). Item difficulty on the pretest was assessed. Twenty-five of 37 (68%) questions had 75% or more of respondents answering correctly and 11 of 37 (30%) had between 75% and 25% answering correctly. For the 37 questions, the median percentage of residents that answered the questions correctly was 82% (IQR: 65 - 99). This suggests that the questions skewed toward a low level of difficulty.

Pre- and post-evaluation survey data were available for 57 participants for a response rate of 80%. Sixty percent (95% CI: 46, 72) of residents reported completion of < 25 ultrasound examinations prior to training, 13% (CI: 4, 22) had completed 26 - 50 examinations, 4% (CI: 1, 13) had completed 76-100, and 23% (CI: 14, 36) had completed greater than 100 examinations. The number of examinations performed prior to the course was not related to the year of the session (p = 0.67).

Residents reported that prior to the introductory ultrasound course, they were most confident performing a FAST examination, reporting a median of 5.5 (IQR: 3 - 7) on a 10-point Likert scale where 1 represented low confidence and 10 represented high confidence. They reported the lowest confidence in performing cardiac ultrasound 3 (IQR: 2 - 6). Following training, residents reported increased confidence with all applications (p < 0.001). Table 1 shows the median confidence for each examination before and after the orientation, along with the median increase in confidence.

**Table 1:** Confidence in Performing Ultrasound Examination Confidence of postgraduate year-1 emergency medicine residents in performing various ultrasound examinations before and after a one day training session ^†^(1 = lowest confidence, 10 = highest confidence) *p < 0.0001, Wilcoxon matched-pairs signed-rank test FAST: focused assessment with sonography for trauma

	Confidence in Performing Ultrasound Examination^†^		
	Median (Interquartile Range)		
Ultrasound Examination	Before Orientation	After Orientation	Increase
FAST	5.5 (3 - 7)	7.5 (6 - 9)	2 (1 - 3)*
Aorta	4 (2 - 6)	7 (5 - 8)	2.5 (1 - 4)*
Renal	4 (2 - 6)	7 (6 - 8)	2 (1 - 4)*
Cardiac	3 (2 - 6)	7 (5 - 8)	2.5 (1 - 4)*
Soft Tissue	5 (3 - 7)	7.5 (5 - 9)	2 (1 - 4)*
Thoracic	3.5 (2 - 7)	7 (6 - 8)	3 (1 - 5)*
Vascular Access	5 (2 - 7)	8 (6 - 8)	2 (1 - 4)*

The percentage of residents reporting that learning objectives were met with asynchronous curriculum was as follows: demonstrating techniques required to perform different types of ultrasound examinations - 96% (CI: 86, 99); describing sonographic appearance of common pathologies - 100% (CI: 94, 100); outlining steps for successful ultrasound-guided vascular access - 100% (CI: 94, 100); and how to dynamically track needles using ultrasound guidance - 100% (CI: 94, 100). The percentage of residents that agreed or strongly agreed with various statements about the ultrasound orientation was as follows: “Online ultrasound lectures effectively teach point-of-care ultrasound applications” - 85% (CI: 73, 92) and “Case-based interactive sessions helped me understand how ultrasound changes the management of acutely ill patients in the emergency department" - 98% (CI: 88, 100).

## Discussion

Tasked with educating all residents to the same level of competency, we have developed an introductory ultrasound course that combines asynchronous learning, case-based interactive sessions, goal-directed, and hands-on training sessions, as well as written and skills assessments. Our experience is unique in that we sought to develop a curriculum for ultrasound orientation at the beginning of residency with the end of goal of developing residents who are highly skilled at the clinical integration of ultrasound into patient management while maximizing resources.

We chose a flipped classroom style of instruction to optimize in-classroom and didactic time. In a flipped classroom, also called asynchronous learning, information that would usually be provided as a didactic or lecture is provided for the learner to review outside of classroom time. The classroom time is then used for teaching practical implementation of skills, stimulating discussions between teacher and learner, and assessment of understanding. Online medical educational resources abound and the use of these resources to deliver information are growing in many areas of education, including graduate medical education [17]. Initial studies show that this method of education is well-received, particularly by younger learners [18]. There is some evidence that asynchronous learning is effective in teaching some content, but that traditional didactic lectures may be optimal for advanced topics [19-20]. In this study, residents felt as though online lectures effectively taught emergency ultrasound applications. Asynchronous learning may allow residents an opportunity to self-educate on the topics in which they felt the most deficient. Self-assessment of skills and personal tailoring of educational needs may not always be accurate, however.

Interactive case sessions were integrated into the introductory ultrasound course in order to highlight the clinical value of point-of-care ultrasound in emergency department patient care. Residents are required to demonstrate integration ultrasound applications into clinical management prior to completion of residency [2]. Educating residents in thoughtful clinical integration from the beginning of residency prepares them to achieve competency prior to graduation. Clinical shifts contribute more to resident educational gains more than dedicated ultrasound rotations, likely because of the clinical integration and emphasis on resuscitative and procedural ultrasound [21]. Residents consider a procedural and resuscitative ultrasound to be more important to their future practice than diagnostic ultrasound [22]. With an emphasis on clinical integration rather than image acquisition early in residency, we can capitalize on the learning that occurs during residents’ clinical emergency department shifts.

Goal-directed, checklist-oriented, hands-on sessions were chosen to deliver uniform education. In skills assessments in simulation training, checklists are commonplace and have good inter-rater reliability and correlation with global assessments [23]. This method decreases variability between educators and diminishes personal bias or educational deficiencies present in the educators. We did not stratify learners based on their educational level, though it may be advantageous to tailor sessions based on prior ultrasound experience. Accounting for the current existing diversity in medical school ultrasound education may optimize the educational experience so that no learners are bored or overwhelmed. If advanced learners could spend less time covering the FAST examination in the hands-on session, they could potentially gain more learning points on other applications. However, there is a risk of error in self-assessment, where some could underestimate their skills and others gravitate toward a review of comfortable applications. Ideally, residents would be stratified based on initial assessments prior to the session. Since we found that a written test of knowledge regarding the use of point-of-care ultrasound does not correlate with procedural skills at the start of residency, evaluation of both skills sets may be necessary to tailor the training.

Limitations

There are inherent limitations to this study. First, residents were not reassessed to see if their new knowledge and skills remained consistent throughout the year. We developed no markers to assess for the effect of ultrasound orientation on meeting the emergency medicine milestones. We did not have a system to ensure the residents reviewed the online lectures. We also noted variation among the faculty assessors, which could have been addressed with additional preparation of the assessors. Residents self-reported their confidence levels, and this could have been better assessed with identical assessments prior to and after the introductory ultrasound course.  

## Conclusions

Ultrasound orientation as part of the introduction to residency is recommended and is nearly ubiquitous in emergency medicine residency programs. This study demonstrates that residents have increased confidence in performing the ultrasound applications taught during a multimodular introductory ultrasound course and that educational objectives can be met with asynchronous learning techniques. Further study is needed to address the optimal methods and assessments that should take place as part of ultrasound orientation for a diverse group of interns.
